# Spectrum of perforation peritonitis in India-review of 504 consecutive cases

**DOI:** 10.1186/1749-7922-1-26

**Published:** 2006-09-05

**Authors:** Rajender Singh Jhobta, Ashok Kumar Attri, Robin Kaushik, Rajeev Sharma, Anupam Jhobta

**Affiliations:** 1 Department of General Surgery, Government Medical College and Hospital (GMCH), Chandigarh (Pin: 160047), India

## Abstract

**Background/objective:**

Perforation peritonitis is the most common surgical emergency in India. The spectrum of etiology of perforation in Tropical countries continues to be different from its Western counterpart. The **objective **of the study was to highlight the spectrum of perforation peritonitis as encountered by us at Government Medical College and Hospital (GMCH), Chandigarh.

**Methods:**

Five hundred and four consecutive cases of perforation peritonitis over a period of five years were reviewed in terms of clinical presentation, operative findings and postoperative course retrospectively at GMCH, Chandigarh.

**Results:**

The most common cause of perforation in our series was perforated duodenal ulcer (289 cases) followed by appendicitis (59 cases), gastrointestinal perforation due to blunt trauma abdomen (45 cases), typhoid fever (41 cases) and tuberculosis (20 cases). Despite delay in seeking medical treatment (53%), the overall mortality (10%) was favourably comparable with other published series though the overall morbidity (50%) was unusually high.

**Conclusion:**

In contrast to western literature, where lower gastrointestinal tract perforations predominate, upper gastrointestinal tract perforations constitute the majority of cases in India. The increasing incidence of post-traumatic gastro-enteric injuries may be due to an increase in high speed motor vehicle accidents which warrant early recognition and prompt treatment to avoid serious complications and death.

## Background

Perforation peritonitis is the most common surgical emergency in India. Despite advances in surgical techniques, antimicrobial therapy and intensive care support, management of peritonitis continues to be highly demanding, difficult and complex. The spectrum of etiology of perforation continues to be different from that of western countries[1] and there is paucity of data from India regarding it's etiology, prognostic indicators, morbidity and mortality patterns[2]. Our study was designed to highlight the spectrum of perforation peritonitis as encountered by us at Government Medical College and Hospital (GMCH) Chandigarh.

## Patients and methods

A retrospective analysis of 504 patients of perforation peritonitis was done over a period of last five years at GMCH Chandigarh.

### Inclusion criteria

All cases found to have peritonitis as a result of perforation of any part of gastrointestinal tract at the time of surgery were included in the study.

### Exclusion criteria

All cases with either primary peritonitis or that due to anastomotic dehiscence were excluded.

All cases were studied in term of clinical presentation, radiological investigations done, operative findings and postoperative course. Data was colleted from indoor patient records, operation theatre records and outpatient department follow up of cases.

All patients following a clinical diagnosis of perforation peritonitis and adequate resuscitation, underwent exploratory laparotomy in emergency setting. At surgery the source of contamination was sought for and controlled. The peritoneal cavity was irrigated with 5–6 litres of warm normal saline and the decision to insert a drain was left to the discretion of the operating surgeon. Abdomen was closed with continuous, number one non-absorbable suture material. Although all patients received appropriate perioperative broad spectrum antibiotics, the drug regimen was not uniform.

## Results

A total of 504 patients were studied. Mean age was 36.8 years(range from 3 to 90 years) with majority of patients being males(84%), 16% were in the age group of more than 50 years and 24% of the patients had atleast one pre-existing medical illness (Table 1).

**Table 1:** Preoperative Data

**Parameter**	**(n = 504)**
**Age (Years)**	
<50 Years	422 (84)
>50 Years	82(16)
**Sex**	
Male	423(84)
Female	82(16)
**Pre existing comorbid conditions**	
Respiratory disease	51 (10)
Renal Disease	27 (50)
Malignancy	25 (5)
Hypertension	11 92)
Diabetes mellitus	5 (1)
**Signs and symptoms**	
Pain	495 (98)
Vomiting	296 (59)
Abdominal distention	221 (44)
Constipation	193 (38)
Fever	124 (25)
Diarrhoea	35 (7)
Tachycardia (pulse >/110/minute)	115 (23)
Hypotention (Systolic blood pressure <100 mm Hg)	44 (9)
Urine output (<30 ml/hr)	80 (16)
Tacchypnea (respiratory rate >20/minute)	334 (66)
**Investigations**	
Pneumoperitoneum on Chest X-Ray	340(67)
Air fluid levels on abdominal X-Ray	108(21)
Hyponatraemia(Na<130 mEq/L)	148(29)
Hypokalemia(K<2.7 mEq/L)	44(09)
Blood Urea Nitrogen(>167 mg/dl)	76(15)
Serum Creatinine(<1.7 mg/dl)	65(13)

Values in parenthesis are percentages.

The time taken by the patient between onset of symptoms and presentation to the hospital was less than 24 hours in 235(47%) cases and more than 24 hours in 269(53%) cases. The time taken for resuscitation, diagnosis and preparation of patient for surgery was less than 12 hours in 396(79%) and more than 12 hours in 108(21%) patints.

The clinical presentation of the patients varied according to the site of perforation (Table 1). The patint of duodenal ulcer perforation usually had a short history of pain starting in epigastrium or upper abdomen along with generalized tenderness and guarding. 13% of patints had positive history of NSAID consumption.

The patients with small bowel perforation presented with prolonged history of fever followed by the appearance of pain in lower abdomen. Abdominal distention was found in 68% along with vomiting in 60% and constipation in 41% cases. 15% of the patients were in shock at the time of admission. Only 55% had evidence of pneumoperitoneum on chest X-Ray done in erect posture.

Appendicular perforations had characteristic pain starting in the periumbilical area or right iliac fossa along with vomiting(66%) and fever(43%). They had localized guarding(77%) or rebound tenderness in right iliac fossa(68%). Perrectal digital examination showed tenderness in 54% cases. None of the patients of appendicular perforation showed evidence of gas under diaphragm on erect chest X-Ray.

Acid peptic disease was the most common cause of gastroduodenal perforation(90%) whereas typhoid fever was the most common cause of small bowel perforation(45%) followed by tuberculosis(22%) and trauma(15%) (Table 2).

**Table 2:** Operative data

**Parameter**	**(n-504)**
**Site of perforation **(n = 504)	
Duodenal	289(57)
Gastric	42(8)
Jejunal	16(3)
Ileal	76(15)
Appendicular	59(12)
Colonic	19(4)
Esophageal	03(0.5)
**Etiology Gastroduodenal**(n = 331)	
Acid peptic disease	297(90)
Trauma	21(6)
Malignancy	13(4)
**Small bowel **(n = 92)	
Typhoid	41(45)
Tuberculosis	20(22)
Traumatic	14(15)
Malignancy	5(5)
Stiangulation of bowel	5(5)
Unknown etiology	6(7)
**Large boweln **(n = 19)	
Trauma	10(53)
Malignancy	5(26)
Sigmoid volvulus	4(21)
**Type of peritonitis(**n = 504)	
Localised	84(17)
Generalised	420(83)
**Nature of exudates(n **= 504)	
Clear	78(15)
Purulent	358(71)
Fecal	68(13)
**Surgical procedure **(n = 504)	
Simple closure	304(60)
Resection with anastomosis*	46(9)
Resection without anastomosis*	64(13)
Definitive procedure^$^	33(6)
Appendicectomy	57(11)

Values in parethesis are percentages

*Ileostomy/colostomy with mucus fistula/Hartman's procedure

^$^Bilroth 1/Bilroth11/Truncal vagotomy and drainage proceedure

In majority of patients(83%) the peritonitis was generalized and the contamination was either purulent or fecal(84%). The other operative findings and surgical procedures performed are as illustrated in Table 2.

251 of 504 cases incurred postoperative complications (Table 3). The morbidity rate in our study was significantly higher in the patients with intestinal perforation(68%) than those with gastroduodenal perforation(47%). In perforated small bowel patients, the presenting complaints were higher in contrast to the patients of gastroduodenal perforation.

**Table 3:** Postoperative Complications

**Complication**	**n = 504**
Abdominal collection	46(9)
Wound infection	126(25)
Pneumonia	143(28)
Dyselectrolaemia	88(17)
Septicemia	90(18)
Acute renal failure	51(10)
Burst abdomen	44(9)
Anastomotic leak	34(7)
Overall morbidity	251(50)
Mortality	51(10)

Values in parenthesis are percentages.

The overall mortality rate in our study was 10% (Table 3) with septicemia associated with MOSF being the most common cause of death in 30 cases(59%) followed by respiratory complications in 12(20%), acute myocardial infarction in 3(6%), pulmonary embolism in 2(4%) and anastomotic leak in 4(8%)cases. Factors contributing to mortality were advanced age, perforation presenting after 24 hours and respiratory complications.

## Discussion

Perforation peritonitis is a frequently encountered surgical emergency in tropical countries like India, most commonly affecting young men in the prime of life as compared to the studies in the west[3] where the mean age is between 45–60 years. In majority of cases the presentation to the hospital is late with well established generalized peritonitis with purulent/fecal contamination and varying degree of septicemia. The signs and symptoms are typical and it is possible to make a clinical diagnosis of peritonitis in all patients.

The perforations of proximal gastrointestinal tract were six times as common as perforations of distal gastrointestinal tract as has been noted in earlier studies from India[1], which is in sharp contrast to studies from developed countries like United States[4], Greece[5] and Japan[6] which revealed that distal gastrointestinal tract perforations were more common.

Not only the site but the etiological factors also show a wide geographical variation. Khanna et al[7] from Varanasi studied 204 consecutive cases of gastrointestinal perforation and found that over half(108 cases) were due to typhoid. They also had perforations due to duodenal ulcer(58), appendicitis(9), amoebiasis(8) and tuberculosis(4). These figures show the importance of infection and infestation in the third world which is also reflected in the high incidence of typhoid and tubercular perforation in our study. At the other end of the spectrum, Noon et al[8] from Texas studied 430 patients of gastrointestinal perforation and found 210 cases to be due to penetrating trauma, 92 due to appendicitis and 68 due to peptic ulcer. This shows the importance of trauma in developed countries. However, the increased incidence of gastrointestinal perforations due to blunt trauma in the present series(9%) and 21% in another study by Bose et al[9] from PGIMER Chandigarh, may be due to high speed road traffic accidents on national highway near Chandigarh.

Duodenal to gastric ulcer ratio was 7:1 in the present series and 15:1 noted in an earlier study from India[1]. Contrary to this the ratio is 4:1 in studies from United Kingdom[10] and United States[11].

There were 51(10%) deaths within 30 postoperative day which is comparable with other published series [10-12] despite delay in seeking medical treatment. This was probably because of lower mean age(which is a factor determining mortality) of patients in our study. The main cause of death in the present series of patients was septicemia(59%). Therefore contamination is a crucial consideration in patients with peritonitis and problem of mortality is a problem of infection. So by early surgical intervention, we succeed in preventing further contamination by removing the source of infection though the end result will also depend upon the general host resistance and the antibiotic sensitivity of the organism[13].

The major cause of postoperative morbidity were respiratory complications(28%) e.g. pneumonia, atelectasis, pleural effusion or ARDS, wound infection(25%), septicemia(18%) and dyselectrolaemia(17%) which are preventable and should be detected early and aggressively treated. Unacceptably high incidence of abdominal wall disruption(9%) in the present series was multifactorial due to delayed presentation, gross contamination of peritoneal cavity, septicemia and above all the faulty methods of abdominal closure as majority of our patients were operated by inexperienced resident doctors who are a floating population and are still in the learning curve.

To conclude, the spectrum of perforation peritonitis in India continues to be different from its western counterpart with duodenal ulcer perforation, perforating appendicitis, typhoid perforation and tubercular perforation being the major causes of generalized peritonitis. The increasing incidence of hollow visceral injuries due to blunt abdominal trauma is a diagnostic dilemma for the surgeons and warrants early recognition and prompt treatment to avoid major morbidity and mortality.
